# Pharmacological Analysis of the Rat Femoral Artery Response to Bradykinin

**DOI:** 10.3797/scipharm.1305-13

**Published:** 2013-06-04

**Authors:** Miroslav Radenković, Marko Stojanović, Nebojša Skorupan, Milica Prostran

**Affiliations:** Department of Pharmacology, Clinical Pharmacology and Toxicology; Faculty of Medicine; University of Belgrade; PO Box 38; 11129 Belgrade; Serbia.

**Keywords:** Bradykinin, Femoral artery, Endothelium, Ca^2+^ channels, Thromboxane A_2_Introduction

## Abstract

Bradykinin (BK) plays an important role in different physiological processes including the general preservation and modulation of vascular systems. The present study was designed in order to examine the effect of BK on isolated rat femoral artery rings and to investigate the participation of intact endothelium, cyclooxygenase products, Ca^2+^ channels, Na^+^/K^+^–ATPase, and B_2_ kinin receptors in BK-induced action. Circular artery segments were placed in organ baths. The endothelium was mechanically removed from some arteries. Concentration–contraction curves for BK were obtained in the rings previously equilibrated at the basal tone. BK produced a concentration–dependent contraction, which was reduced by endothelial denudation. The BK–induced effect was almost completely inhibited by indomethacin (cyclooxygenase inhibitor) or OKY–046 (thromboxane A_2_–synthase inhibitor). Nifedipine (Ca^2+^ channel blocker), ouabain (Na^+^/K^+^–ATPase inhibitor), or HOE–140 (selective B_2_ kinin receptor antagonist) significantly reduced the BK–evoked effect. In conclusion, it can be proposed that BK produces concentration– and endothelium–dependent contractions of the isolated rat femoral artery, which is for the most part a consequence of B_2_ kinin receptor activation. Cyclooxygenase contractile products, especially thromboxane A_2_, play a significant role in this course of action. The transduction mechanism involved in the process of BK–induced femoral artery contraction include the activation of voltage–gated Ca^2+^ channels, and in a smaller extent Na^+^/K^+^–ATPase as well.

## Introduction

Bradykinin is an endogenous polypeptide, which belongs to the kinin family. It controls and modulates important physiological processes and has been recognized as an exceptionally useful pharmacological tool. Aside from bradykinin, kallidin and methionyl-lysyl-bradykinin also belong to the kinin family, and in plasma they are converted to bradykinin by the enzymatic activity of aminopeptidases [1]. The kinins are generated locally by proteolytic action of kallikreins on kininogen, and they act accordingly as local hormones. However, in less than 15 seconds, kinins are commonly inactivated by circulating kininases [1].

The kinins exert their activity through the selective activation of specific kinin receptors. This group of receptors encompasses two well-defined types, named B_1_ and B_2_ kinin receptors [2, 3]. Two additional kinin receptors have been also proposed, namely B_3_ and B_4_, although these are still under investigation [4, 5]. B_1_ and B_2_ kinin receptors belong to the family of G-protein–coupled receptors (G_q/11_), thus expressing the similar intracellular transduction signaling cascade [6]. B_2_ kinin receptors are defined as important constitutive receptors with the predominant localization in smooth muscle cells and the heart; whereas B_1_ kinin receptors are considered to be inducible receptors, and their expression follows tissue injuries, inflammation, and action of different endogenous factors, including endotoxins, cytokines, or various growth factors [7].

Bradykinin plays an important role in different physiological processes, especially in those associated with general preservation and functioning of the cardiovascular system [8]. Besides the physiological activity, bradykinin is also included in the pathogenesis of different diseases. For example, the pivotal role of bradykinin has been well-studied in the etiology of pain, allergy, rhinitis, fever, and different gastrointestinal pathological processes [7]. Accordingly, after the central and peripheral stimulation of the sympathetic tone, bradykinin may further deteriorate existing hypertension [9]. It has also been established that bradykinin produces protective actions in different pathological conditions such as ischemia/reperfusion injury, as well as in myocardial infarction and cerebral vascular disorders [10, 11].

With consideration of vascular system effects, bradykinin can produce vasoconstriction by the activation of B_2_ kinin receptors located on smooth muscle cells, and oppositely vasodilatation after the activation of B_1_ and endothelial B_2_ kinin receptors. This has been shown to be largely dependent upon the investigated species or/and the chosen blood vessel [8, 12, 13]. Bradykinin-induced vasodilatation is commonly mediated by endothelial relaxing autacoids, especially nitric oxide (NO), while the characterization of bradykinin contractile effects is still an ongoing process with notable implications that different cyclooxygenase products might probably be involved [12].

Taking the previous considerations into account, followed by the known fact that the femoral artery represents an important target site connected to various pathological conditions, and finally that the bradykinin action on this blood vessel is still under investigation, the present isolated organ bath study was undertaken in the following order: (1) to examine the effect of bradykinin on isolated rat femoral artery rings, (2) to investigate the possible participation of intact endothelium in bradykinin-evoked action, (3) to define a possible role of cyclooxygenase products in bradykinin action on the investigated blood vessel, (4) to establish if voltage-gated Ca^2+^ channels and Na^+^/K^+^-ATPase are important for the transduction mechanism related to the bradykinin vascular effect; (5) and finally to determine if B_2_ kinin receptors are involved in bradykinin’s action on the rat femoral artery.

## Material and Methods

### Animals

Our investigation was performed on male rats, but in the initial phase of the experiments, female albino rats of the Wistar strain that were one month-old and 250–280g in weight were used before the beginning of the experiments. The animals were maintained under a standard light/day cycle (12 h light/12 h dark cycle), at adequate temperature (24 ± 1°C) and humidity (55 ± 10%), and were allowed *ad libitum* access to pellets of standard rodent diet as well as tap water.

### Vascular Ring Preparation

The methodology described in this article was in accordance with our previous investigations [8, 14, 15]. The left and right femoral arteries were carefully isolated from rats, dissected from connective and fat tissue, cut into 4mm–long vascular rings and immediately placed in Krebs-Ringer bicarbonate solution. On randomly selected preparations, the endothelium was removed with a stainless–steel wire by gently rubbing the intimal surface. The vascular rings were positioned between two stainless-steel triangles and placed in an organ bath field with Krebs-Ringer bicarbonate solution, aerated with 95% O_2_ and 5% CO_2_ and kept at 37°C and a pH of 7.4. One of the triangles was attached to a displacement unit allowing for fine adjustment of the tension, and further connected to the force-displacement transducer (Hugo Sachs Elektronik F30 Type 372, Freiburg, Germany). Isometric tension was continuously recorded on the Rikadenki R-62 multi-pen electronic recorder (Rikadenki Kogyo CO., LTD, Tokyo, Japan).

### Experimental Protocol

After the initial setup procedure, the preparations were allowed to equilibrate for 45 minutes in an organ bath. Afterwards, each vascular ring was progressively stretched to the optimal resting tension of 1.5 g during the period of 30 minutes. The resting tension corresponding to 1.5 g was previously determined by length-tension relationship experiments. At the beginning of each experiment, the endothelial functional integrity was pharmacologically investigated with the addition of acetylcholine (1 μM) after the contraction of the femoral artery was induced with a submaximal concentration (EC_50_–EC_70_) of phenylephrine. This procedure was performed three times within 20-minute interval in-between. Preparations, in which the obtained relaxation was over 80% of the phenylephrine precontraction, were considered to have a functional endothelium. Cumulative concentration-contraction curves for bradykinin (0.001–0.3 μM) were obtained for rings previously equilibrated at basal tone. The higher concentration of bradykinin was administered to an organ bath only after the equilibrium response to the lower concentration had been produced. One vascular ring served as a time control, and was exposed only to bradykinin, while the other rings from the same animal were treated with the pharmacological blocker of interest 30 minutes before the cumulative addition of bradykinin. This type of experiment was performed because preliminary experiments of the preparations of the femoral artery demonstrated that the first and second concentration-contraction curve (determined 45 min apart) for bradykinin were significantly different. At the end of each experiment, the vascular rings were additionally equilibrated for 15 minutes at the level of the resting tension and finally contracted with 60 mM KCl. The contraction induced by each concentration of bradykinin is expressed as a percentage of the contraction produced by 60 mM KCl (100%), and it was used in the construction of the concentration-response curves. The results were expressed as the means ± S.E.M. and n refers to the number of experiments. The concentration of bradykinin producing 50% of its own maximum response (EC_50_) was determined by using a non-linear least square fitting procedure of the individual experimental data, and was presented as pEC_50_ (pEC_50_ = −log EC_50_).

Apart from the previous protocol, and in order to determine the participation of extracellular Ca^2+^ ions in bradykinin–induced action on the femoral artery, the Krebs–Ringer bicarbonate solution was replaced by an organ bath with the Ca^2+^-free Krebs–Ringer bicarbonate solution. After the replacement of the bath solution, the preparations were allowed to equilibrate for 20 minutes at the resting tension level. This stabilization period was followed by the single addition of a high concentration of bradykinin (10 μM). Taking into account that in the Ca^2+^-free solution, the final KCl (60mM)-induced contractions were inconsistent; the obtained bradykinin-induced contraction was expressed as a percentage of the third phenylephrine-evoked contraction from the initial part of the corresponding experiment.

### Drugs and Solutions

The Krebs–Ringer bicarbonate solution had the following composition (in mM): NaCl 118.3; KCl 4.7; CaCl_2_ 2.5; MgSO_4_ 1.2; KH_2_PO_4_ 1.2; NaHCO_3_ 25.0; Ca-EDTA 0.026; glucose 11.1. Ca^2+^-free Krebs–Ringer bicarbonate solution was prepared by replacing CaCl_2_ with an equimolar concentration of MgCl_2_, also 0.026mM Ca-EDTA was replaced with 0.5 mM EGTA. The following chemicals were used: acetylcholine iodide (Serva, Heidelberg, Germany); bradykinin (ICN, Irvine, CA, USA); D-Arg[Hyp^3^,Thi^5^,D-Tic^7^,Oic^8^]-bradykinin (HOE 140), indomethacin, nifedipine, ouabain, phenylephrine (Sigma –Aldrich, St Louis, USA); (2*E*)-3-[4-(1*H*-imidazol-1-ylmethyl)phenyl]prop-2-enoic acid – OKY-046 (Ono Pharmaceutical, Osaka, Japan). All agents except indomethacin and nifedipine were dissolved in distilled water and diluted to the desired concentration with the buffer. In contrast, indomethacin was prepared in an equimolar Na_2_CO_3_ solution, while nifedipine was diluted in 70% ethanol. Additionaly, indomethacin and nifedipin were diluted to the desired concentration with the buffer. Prepared stock solutions were stored on ice until use. Experiments with ouabain and nifedipine were performed in a dark room. During the experimental procedure all agents were added directly to the bath in a volume of 0.15 ml and the concentrations given are the calculated final concentrations in the bath solution.

### Statistical Analysis

All calculations were done by using the computer program Graph Pad Prism (Graph Pad Software Inc., San Diego, U.S.A.). The results were expressed as means ± S.E.M.. Experimental results were analyzed with Student’s *t*-test and the obtained values were considered to be significant or highly significant versus the control when p values were lower than 0.05 or 0.01, respectively.

## Results

**Fig. 1:**
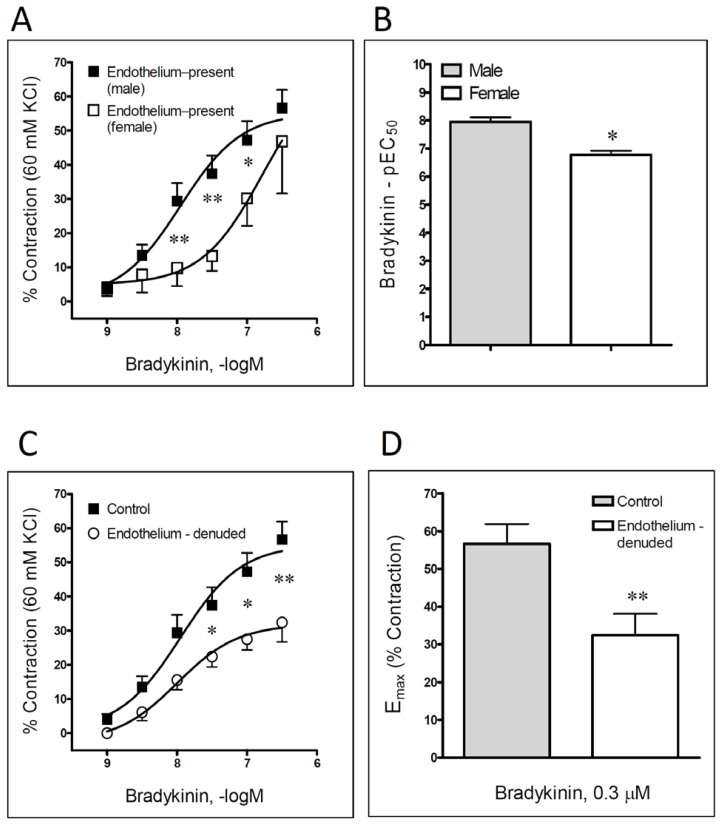
Bradykinin-induced contraction of intact male and female rat femoral artery (A–B), and male femoral artery in the presence and the absence of vascular endothelium (C–D). Concentration of bradykinin eliciting 50% of its own maximum response (EC_50_) is presented as pEC_50_ (pEC_50_ = −log *EC_50_*), whereas maximal obtained contraction (E_max_) is expressed as a percentage of the contraction produced by 60mM KCl. Each point represents the mean ± SEM (n = 4–7). *P < 0.05 and **P < 0.01 compared to the respective control.

Bradykinin (0.001–0.3 μM) produced a concentration-dependent contraction of the isolated rat femoral artery (Table 1, Figure 1A). There was a statistically significant difference in the response to bradykinin in preparations obtained either from male or female rats. Namely, considering the obtained pEC_50_ values, bradykinin produced notably more potent contractions in male rats, which was on the other hand equi-effective, if merely maximal contractile responses were compared (Table 1, Figure 1A–B). Taking into account that there was an obvious influence from gender involved in the vascular response to bradykinin, and in order to reduce the number of animals actually required for our investigation, the further experiments included only rings obtained from the male animals, which was in full accordance with the “The 3Rs” principle [16–18].

**Fig. 2:**
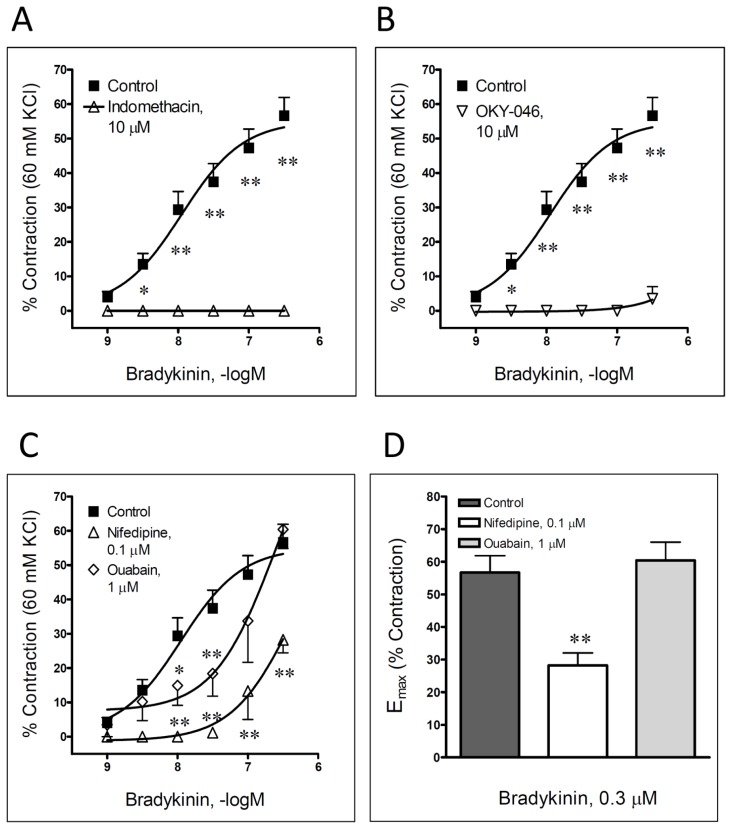
Bradykinin-induced contraction of isolated male rat femoral artery in the absence, or in the presence of indomethacin (A) and OKY-046 (B), as well as nifedipine and ouabain (C–D). Bradykinin-evoked contractions, including maximal obtained contraction (E_max_), are expressed as a percentage of the contraction produced by 60mM KCl. Each point represents the mean ± SEM (n = 4–7). *P < 0.05 and **P < 0.01 compared to the respective control.

After the process of endothelial denudation, bradykinin-induced contraction was notable, yet not entirely reduced (Table 1, Figure 1C–D). On the other hand, an application of indomethacin (a cyclooxygenase inhibitor, 10 μM) completely abolished the bradykinin-produced contraction of the femoral artery (Figure 2A). A comparable result was obtained after the incubation of 10 μM OKY-046, a thromboxane A_2_-synthase inhibitor (Figure 2B).

**Fig. 3:**
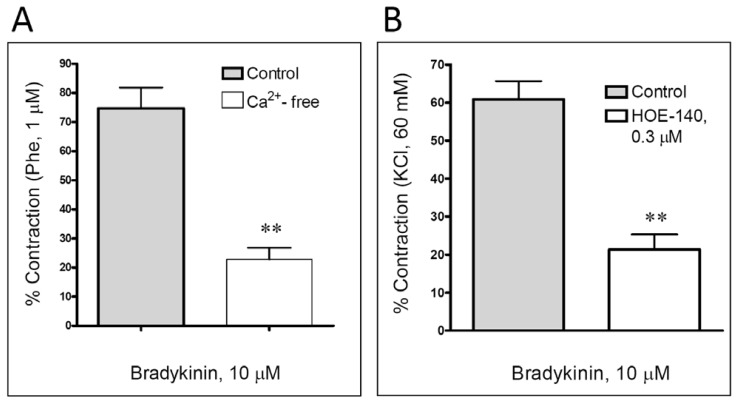
Bradykinin-induced contraction of isolated male rat femoral artery in the Ca^2+^–free solution (A) or in the presence of HOE–140 (B). The obtained contraction is expressed as a percentage of the contraction produced by 1 μM phenylephrine (Phe) (A) or 60 mM KCl (B). Each result represents the mean ± SEM (n = 4–7). **P < 0.01 compared to the respective control.

An application of nifedipine (an L-type voltage-gated Ca^2+^ channel blocker; 0.1 μM) in the first three concentrations completely inhibited the vascular response of the femoral artery to bradykinin (Table 1, Figure 2C–D). Additionally, the replacement of the Krebs–Ringer bicarbonate solution with the Ca^2+^-free solution significantly, still not completely, reduced the contraction of the rat femoral artery induced by a single application of a high concentration of bradykinin – 10 μM (Figure 3A). The addition of ouabain (a Na^+^/K^+^-ATPase inhibitor, 1 μM) in an organ bath resulted in the reduction of contractile effects only in the middle range of the applied bradykinin concentrations (Table 1, Figure 2C–D). In other words, the concentration–dependent curve for bradykinin was shifted to the right with the correspondent reduction of the obtained pEC_50_ value versus the control, while the maximal contraction was comparable regardless of the presence of ouabain.

Finally, HOE 140 (0.3 μM), a selective B_2_ kinin receptor antagonist, significantly reduced the contraction of the femoral artery induced by a single addition of 10 μM bradykinin (Figure 3B).

## Discussion

Bradykinin is a well-characterized peptide, with the potential to modulate vascular tone in different blood vessels. Even though bradykinin is most commonly considered as a vasodilating substance [13, 19], certain studies have stressed its potential in producing contractile effects in the investigated animals and human blood vessels [8, 12, 20]. The results of our experiments have shown that in the rat femoral artery, bradykinin produced a concentration-dependent contraction. The vascular response to the examined kinin was significant, yet not completely reduced after the process of endothelial denudation, thus indicating a partial contribution of the endothelium. In other words, it can be presumed that bradykinin-evoked vascular contraction is probably associated with the release of some endothelial contractile autacoid(s), or oppositely with the plausible inhibition of endothelium-derived relaxing factors. Therefore, it is not surprising that our result is in accordance with the findings obtained in the investigation of bradykinin’s effects in the porcine interlobar renal artery [21], yet still in contrast with those obtained in the rat tail artery [22].

The next part of our investigation was aimed to determine the possible role of arachidonic acid metabolites in bradykinin-produced effects. A number of studies demonstrated that the different cyclooxygenase products of arachidonic acid, such as prostaglandin F_2α_ (PGF_2α_), prostaglandin H_2_ (PGH_2_), or thromboxane A_2_ (TXA_2_), have been involved in the bradykinin contractile effects in various blood vessels [12, 23–27]. Our results were in line with the quoted studies, since cyclooxygenase inhibition completely abolished the bradykinin-produced contraction of the femoral artery. Comparable results were obtained after the inhibition of thromboxane A_2_-synthase, suggesting that thromboxane A_2_ is most probably an arachidonic acid product involved in the transduction mechanism of bradykinin contraction in the investigated blood vessel. Moreover, since cyclooxygenase and thromboxane A_2_-synthase inhibition reduced bradykinin-induced contraction to a higher extent than endothelial denudation itself, it can be assumed that thromboxane A_2_ involved in bradykinin-produced contraction has both endothelial and smooth muscle cell origin. Nevertheless, this result requires additional clarification.

The most important step in any vascular smooth muscle contraction is an increase in the intracellular calcium concentration. The calcium-mediated vascular effects of different vasoactive substances in general involve the activation of calcium channels or/and Na^+^/K^+^-ATPase [8, 14]. In our experiments we have recorded a strong reduction in bradykinin-induced contraction after the voltage-gated L-type Ca^2+^ channels were blocked with nifedipine. Thus, our data indicate that an increase in intracellular calcium concentration via L-type voltage-gated Ca^2+^ channels significantly contributes to the bradykinin-mediated vasoconstriction in the rat femoral artery. This was also confirmed by additional experiments in which a significant reduction in bradykinin-produced contractions in Ca^2+^-free solution was traced. Accordingly, the importance of extracellular calcium in the contractile effects of bradykinin was previously confirmed in the porcine interlobar renal artery [21].

Apart from the Ca^2+^ channel activation, an increase in the intracellular calcium concentration can be also achieved *via* modulation of Na^+^/K^+^-ATPase. In our investigation, a Na^+^/K^+^-ATPase inhibition significantly, yet partly, reduced the pharmacological potency without affecting the pharmacological efficacy of the femoral artery response to bradykinin. Based on this result it can be presumed that the bradykinin- elicited contractile effect in the rat femoral artery is only partly due to the activation of Na^+^/K^+^-ATPase and a consequent increase in intracellular calcium. Although we could not find corresponding data in the literature regarding the role of Na^+^/K^+^-ATPase in bradykinin-produced vascular contractions, Dodson *et al*. [28] previously confirmed the increased activity of Na^+^/K^+^-ATPase in guinea pig tracheal smooth muscle cells as a result of bradykinin’s action.

Bradykinin produces various pharmacological effects through the selective activation of pharmacologically distinct B_1_ and B_2_ kinin receptors. The B_2_ kinin receptors are ubiquitous and constitutively expressed, while the B_1_ kinin receptors are poorly expressed in healthy tissues, which is commonly changed during non-specific tissue injury and inflammation. Previous findings have suggested that bradykinin-produced vascular contractile effects were mediated through the activation of B_2_ kinin receptors [8, 26]. Thus, in the final part of our investigation, we have examined the role of B_2_ receptors in the bradykinin-induced contraction of the isolated rat femoral artery by challenging the artery segments with HOE 140, a selective antagonist of B_2_ kinin receptors. As presumed, bradykinin-induced contraction was strongly reduced, thus indicating a notable contribution of B_2_ kinin receptors in this process. Taking into account our results on endothelium-present and endothelium-denuded vascular segments, it is plausible to suggest that B_2_ kinin receptors are both present in endothelial and smooth muscle cells. Still, this assumption requires further experimental evaluation.

In conclusion, it can be proposed that bradykinin produces concentration- and endothelium-dependant contraction of the isolated rat femoral artery, which is for the most part a consequence of B_2_ kinin receptor activation. Cyclooxygenase contractile products, especially thromboxane A_2_, play a significant role in this course of action. The transduction mechanism involved in the process of bradykinin-induced femoral artery contraction include the activation of L-type voltage-gated Ca^2+^ channels, and in a smaller extent Na^+^/K^+^-ATPase as well. Taking into account that femoral arteries have a pivotal role in supplying the lower limbs with oxygenated blood, and that alteration in the morphological or/and functional integrity of these vessels can ultimately decrease blood flow to skeletal muscles, it can be proposed that the presented investigation provides an additional contribution to overall knowledge in regard to the pharmacological mechanisms included in the femoral artery response to bradykinin. This can also be relevant in regard to the continuing increase in reported clinical cases involving atherosclerosis, peripheral artery disease, and intermittent claudications that are related to an altered response of endothelial and smooth muscle cells in femoral arteries to different vasoactive agents.

## Figures and Tables

**Tab. 1:** Bradykinin-induced contraction of isolated male rat femoral artery in the absence, or in the presence of specific pharmacological intervention. Concentration of bradykinin eliciting 50% of its own maximum response (EC_50_) is presented as pEC_50_ (pEC_50_ = −log *EC_50_*), whereas maximal obtained contraction (E_max_) is expressed as a percentage of the contraction produced by 60mM KCl. Each result represents the mean ± SEM (n = 4–7).

Pharmacological intervention	pEC_50_ ± SEM	E_max_ (%) ± SEM
Control/Endothelium-present	7.95 ± 0.16	56.7 ± 5.3
Endothelium-present (female)	6.78 ± 0.14*	46.9 ± 15.2
Endothelium – denuded	7.99 ± 0.11	32.4 ± 5.7^**^
Nifedipine (0.1 μM)	6.36 ± 0.28*	28.3 ± 3.8^**^
Ouabain (1 μM)	6.62 ± 0.22*	60.4 ± 5.6

^*^P < 0.05 and ^**^P < 0.01 compared to the respective control.
